# Traditional and Emerging Biomarkers in Asymptomatic Left Ventricular Dysfunction—Promising Non-Coding RNAs and Exosomes as Biomarkers in Early Phases of Cardiac Damage

**DOI:** 10.3390/ijms22094937

**Published:** 2021-05-06

**Authors:** Milijana Janjusevic, Alessandra Lucia Fluca, Federico Ferro, Giulia Gagno, Yuri D’Alessandra, Antonio Paolo Beltrami, Gianfranco Sinagra, Aneta Aleksova

**Affiliations:** 1 Cardiothoracovascular Department, Azienda Sanitaria Universitaria Giuliano Isontina (ASUGI) and Department of Medical Surgical and Health Science, University of Trieste, 34149 Trieste, Italy; mjanjusevic@units.it (M.J.); alessandralucia.fluca@units.it (A.L.F.); fferro@units.it (F.F.); gagnogiulia@gmail.com (G.G.); gianfranco.sinagra@asugi.sanita.fvg.it (G.S.); 2 Cardiovascular Proteomics Unit, Centro Cardiologico Monzino-IRCCS, Via Parea 4, 20138 Milan, Italy; yuri.dalessandra@cardiologicomonzino.it; 3 Department of Medicine (DAME), University of Udine, 33100 Udine, Italy; antonio.beltrami@uniud.it

**Keywords:** asymptomatic heart failure, apparently healed patients, cardiovascular diseases, biomarkers, diagnostic, long non-coding RNAs, miRNA, exosomes

## Abstract

Heart failure (HF) is one of the major causes of morbidity and mortality worldwide and represents an escalating problem for healthcare systems. The identification of asymptomatic patients with underlying cardiac subclinical disease would create an opportunity for early intervention and prevention of symptomatic HF. Traditional biomarkers are very useful as diagnostic and prognostic tools in the cardiovascular field; however, their application is usually limited to overt cardiac disease. On the other hand, a growing number of studies is investigating the diagnostic and prognostic potential of new biomarkers, such as micro-RNAs (miRNA), long non-coding RNAs, and exosome cargo, because of their involvement in the early phases of cardiac dysfunction. Unfortunately, their use in asymptomatic phases remains a distant goal. The aim of this review is to gather the current knowledge of old and novel biomarkers in the early diagnosis of cardiac dysfunction in asymptomatic individuals.

## 1. Introduction

Heart failure (HF) is one of the leading causes of morbidity and mortality, affecting over 64 million people worldwide and representing an economic burden for healthcare systems [1]. Despite the immense progress that has been made in the cardiology field, the total number of patients with HF continues to increase alarmingly due to the growing prevalence of risk factors as well as population aging [2]. Therefore, it is vitally important for clinicians to be able to accurately identify individuals at risk of HF prior to the onset of symptoms.

The prevalence of asymptomatic left ventricular (LV) dysfunction in the general population is estimated between 1% and 4%. These percentages remain debated, raising up to 15% within the high-risk population [3]. The diagnosis of HF in asymptomatic subjects usually occurs during medical consultation for other reasons. Unfortunately, in most cases, due to the scarce adherence of asymptomatic individuals to health screening programs, the diagnosis is delayed towards overt HF, when symptoms become apparent. On the other hand, people with a high risk of cardiac diseases, such as individuals with a genetic predisposition, high blood pressure, or high levels of cholesterol, as well as cancers survivors, are more likely to be early diagnosed with HF because of the frequent check-ups they usually undergo [4]. Beside the above-mentioned risk factors, myocardial infarction (MI) remains the main cause of HF. The loss of myocardial tissue impairs the cardiac contractile function and triggers cardiac remodeling, which is characterized by hypertrophy and fibrosis. Interestingly, only about 25% of patients hospitalized for MI show signs or symptoms of HF, thus making the discovery of biomarkers capable of identifying early asymptomatic LV dysfunction an urgent need [5].

Along with asymptomatic patients, it is important to mention individuals classified as New York Heart Association (NYHA) I. These patients are already diagnosed with HF without symptoms or limitations to their physical activity.

Unfortunately, despite optimal neurohormonal treatment, over the time, these patients experience a progressive worsening of their condition, and therefore the prompt identification of the subset of individuals with a poorer prognosis is of the utmost importance.

Finally, the asymptomatic population with LV dysfunction includes the subset of “apparently healed” patients, i.e., patients diagnosed with HF whose clinical conditions ameliorated after initiating pharmacological therapy. Among “apparently healed” patients diagnosed with HF, molecular changes are suggestive of a less pathological condition, although the heart does not return to a healthy state [6,7]. For instance, several pieces of evidence suggest that in patients affected by idiopathic dilated cardiomyopathy (DCM), the recovery is only temporary [8]. Moreover, symptoms can appear again following the withdrawal from pharmacological treatment [9]. Furthermore, even with therapy adherence, the clinical state tends to worsen over time after an initial stabilization, proving the necessity of a mandatory follow-up [8,10]. 

In the current situation, there is an urgent need for new biomarkers able to indicate the increased probability of HF development and to unveil the undergoing processes responsible for the deterioration of the cardiac function at its very beginning. Furthermore, in patients with “apparent healing”, biomarkers should be able to discriminate recovery from complete remission. The identification of such biomarkers would certainly create an opportunity for an early treatment as well as for new therapeutic targets and enhance the progress towards personalized medicine [11].

This review gathers the current knowledge of the biomarkers used in clinical practice and explores new markers, such as non-coding RNA and exosomes cargo, which may help in HF diagnosis in the asymptomatic phase.

## 2. Current and Emerging Protein Biomarkers

**Figure 1:**
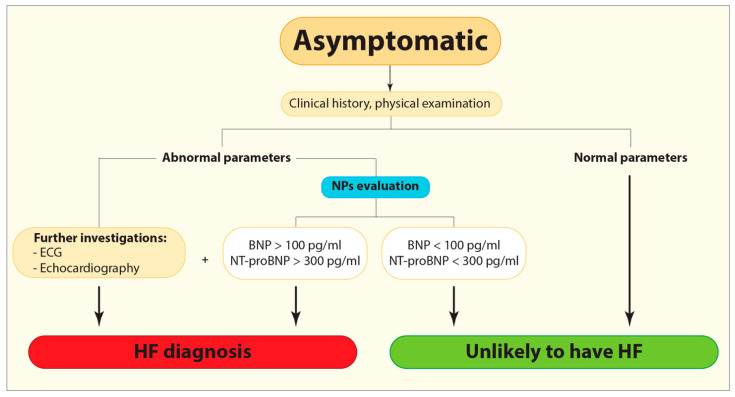
Flow diagram demonstrating the current diagnostic algorithm for HF identification. BNP, B-type or brain natriuretic peptide; ECG, Electrocardiogram; HF, Heart failure; NPs, Natriuretic peptides; NT-proBNP, N-terminal pro-B-type natriuretic peptide.

To date, HF is usually identified when clinical symptoms are apparent [12]. Natriuretic peptides (NPs), such as B-type or brain natriuretic peptide (BNP) and N-terminal pro-B-type natriuretic peptide (NT-proBNP), are traditional cardiac biomarkers in use for distinguishing individuals requiring further clinical investigation, as electrocardiogram and echocardiography, from those who do not (Figure 1) [12]. Specifically, BNP level above 100 pg/mL and NT-proBNP level above 300 pg/mL are the suggested cut-offs for ruling HF in [13]. Noteworthy, the concentrations of BNP and atrial natriuretic peptide (ANP) in asymptomatic individuals with HF are often higher than in healthy controls, but still lower than in symptomatic HF [14]. Moreover, the concentration of NT-proBNP was high among asymptomatic patients with LV dysfunction including those with other comorbidities usually associated with cardiac issues, such as diabetes or peripheral and cerebrovascular diseases [14]. However, caution is needed for using NPs as diagnostic markers, because their concentration varies depending also on other factors including age, body mass index, or the presence of other conditions such as renal failure or inflammatory pulmonary disease [10].

Cardiac troponins (cTn) are a family of proteins widely accepted as the gold standard for acute myocardial infarction (AMI), in particular cTnI and cTnT [13,15]. cTn levels were elevated not only in patients after AMI, but also in patients with acute and chronic HF and are associated with the worst prognosis. Worth mentioning, the increased cTn levels predict adverse events in HF with both preserved and reduced ejection fraction [16,17,18]. Beside the role in the diagnosis of AMI [13], cTn can also be elevated independently of the ischemic etiology of myocardial injury. The detection of cTn among the general population elicited interest in this protein as an indicator of myocardial injury also in asymptomatic individuals [19]. Since its plasmatic concentration in a healthy population is low, the ability to detect cTn is mainly dependent on analytical sensitivity, which is provided by high-sensitivity (hs) assays currently required in clinical practice [20]. Data from the currently available literature indicate that both cTnI and cTnT are detectable in the general population, and it is suggested that an increment in cTn levels could be linked to hypertension, diabetes mellitus, metabolic syndromes, hypercholesterolemia, and genetics [19,20,21]. Interestingly, although the association of cTn with LV hypertrophy and underlying HF has been established, the definition of universal cut-offs among asymptomatic individuals might be challenging due to the influence of age, gender, body mass index, and systolic pressure on cTnI levels and the effect of diabetes mellitus on cTnT [19,20,22]. Therefore, the lack of a clear cut-off might hamper cTn translation to clinical use in the asymptomatic population. In parallel, the definition of the frequency cTn plasmatic concentration assessment and of the characteristics of the target population needs also to be pursued [20].

Emerging biomarkers such as soluble suppression of tumorigenicity 2 (sST2), Galectin-3 (Gal-3), and Ghrelin, reflecting different pathophysiological processes closely associated with fibrosis, appear to have a valuable diagnostic and prognostic power [19]. sST2 levels have been found significantly higher among patients with HF compared to healthy controls, providing insights into disease severity and predicting the risk of future HF events and mortality, independently of NPs levels [19]. Wang et al. were the first to reveal the prognostic value of sST2 measurements in the general population, demonstrating that some asymptomatic individuals could have higher levels of sST2, thereby suggesting the association of this marker with the presence of undergoing processes that could lead to adverse outcomes [23]. Furthermore, given its lower biological variation compared to NPs and the fact that its increase can be observed several weeks before a cardiac event, sST2 is a suitable marker for the follow-up of patients with HF [24,25].

Gal-3 levels have been shown to increase in pre-symptomatic HF animal models as well as during HF progression in both animal models and humans [26,27]. Nevertheless, the use of Gal-3 as a biomarker for asymptomatic LV dysfunction is still debated, mainly due to its low specificity [27]. In fact, because Gal-3 is not organ-specific, the increase of its concentration in plasma could be the consequence of several inflammatory and fibrotic processes in organs other than the heart. 

Finally, studies indicate that the interaction of Ghrelin with its receptor differs between early and end-stage HF [28,29]. In a previous study, we showed that Ghrelin levels were reduced in patients with DCM in comparison with healthy controls [29]. However, among DCM patients, we observed that early diagnosed patients had higher Ghrelin levels than patients with longer duration of the disease [26]. Furthermore, Ghrelin plasmatic levels were found to be higher in the presence of a more compromised LV function, probably due to a compensatory mechanism [29]. Indeed, Ghrelin possesses documented properties improving cardiac function in HF [30]. In addition, it has been reported that changes in Ghrelin myocardial axis could be detectable even before overt changes in LV function, thus supporting its role as a biomarker [28].

Given the fact that biomarkers report on a multitude of harmful pathophysiological processes associated with HF, control screening for NPs and troponin could possibly allow the timely identification of asymptomatic patients at high risk of developing HF [13,14,31]. Furthermore, since asymptomatic subjects (population at high risk of LV dysfunction, “apparently healed” patients with DCM, as well patients with HF in NYHA class I) progress overtime and develop symptoms of overt HF, it would be of paramount importance to assess the concentration of biomarkers and define cut-offs values that would indicate undergoing processes such as biomechanical stress, hypertrophy, and fibrosis that further lead to the deterioration of LV function and overt HF. Recognition of those processes before overt symptoms would be a tremendous achievement. In this context, elevated levels of sST2 and Gal-3 could be “red flags” of ongoing hemodynamic stress and fibrotic processes. sST2 could be an ideal biomarker for monitoring NYHA I patients and individuals at high risk of HF, providing insights into underlying mechanical strain, inflammation, and fibrosis that could later lead to worsening outcomes. In the same context, given its role in cardiac remodeling, Gal-3 elevated concentrations could indicate a worsening of the cardiac condition. Therefore, periodical measurement of Gal-3 should be mandatory during follow-up. Table 1 summarizes the advantages and disadvantages of classical biomarkers in use in the cardiovascular field, as well as those of emerging ones. 

## 3. Non-Coding RNAs

The 90% of the human genome is transcribed in non-coding RNAs, which are classified according to their length in microRNAs (miRNA) and long non-coding RNAs (lncRNA) [32]. Both of them have recently been identified as contributors to HF development [32,33,34].

### 3.1. microRNAs

Nowadays, it is widely accepted that circulating miRNA expression profiles can provide new insights in predicting HF among asymptomatic patients. MiRNAs are short, single-stranded, non-coding RNA molecules, packaged into membranous vesicles such as exosomes, microvesicles, and apoptotic bodies, playing a major role in negative post-transcriptional regulation of gene expression [33,34]. Given their presence and high stability in human fluids such as plasma and serum, they are attractive candidates for disease identification and monitoring [33,35]. Multiple studies have identified cardiac-specific miRNAs involved in cardiac development and function, whereas dynamic changes of cardiac miRNAs have been recognized in pathophysiologic heart conditions. Thus, their diagnostic potential as biomarkers in cardiovascular morbidity and mortality is under intensive investigation nowadays [36,37,38]. 

Despite the identification and thorough investigation of a large number of miRNAs involved in heart remodeling, the use of those miRNAs as indicators of the onset of cardiac changes in asymptomatic individuals remains a rather distant goal and represents an important unmet clinical need. Numerous miRNAs play a key role in the regulation of the processes underlying LV diastolic dysfunction such as fibrosis, hypertrophy, and ischemia. For instance, miR-1 and miR-133a, which are known to have a protective role against cardiac hypertrophy, are downregulated in hypertrophic heart, whereas miR-155, which promotes pro-hypertrophic pathways, is upregulated in the same condition [39]. In the same context, increased expression of miR-21 has been recognized as responsible for fibroblast activation and development of fibrosis [37]. Interestingly, a recent study involving asymptomatic patients with type II diabetes demonstrated that miR-21 levels were significantly decreased in patients with cardiac dysfunction in comparison with those without [40]. Given that diabetic cardiomyopathy affects 12% of diabetic patients and currently lacks a specific biomarker, the opposite behavior of miR-21 in the general and the diabetic populations has important potential implications in the clinical setting [40]. Further, supporting the therapeutic potential of miR-21 as a cardiac biomarker, Tao et al. reported that overexpression of miR-21 improved mitochondrial biogenesis and reduced cell apoptosis in animal models [40].

Research performed by D’Alessandra et al., including healthy individuals and patients with chronic heart failure (CHF), demonstrated that a combined score based on the expression profile of miR-221, miR-21, miR-409-5p, miR-376a, and miR-154 has the highest discrimination power in terms of separating early stage asymptomatic patients with HF (NYHA class I) from healthy controls [41]. Another study proposed miR-499 as a candidate marker for early diagnosis of AMI, showing that the plasma concentration of miR-499 in patients with AMI was significantly higher in comparison with that in healthy controls and correlated with cTnI [42].

miR-624 and miR-340 were significantly upregulated in patients with premature coronary artery disease (CAD) when compared with healthy controls, supporting the use of specific miRNAs for early detection of asymptomatic CAD [43]. Since CAD can lead to heart failure over time, its timely identification would have a relevant impact on the clinical management of patients with CAD. Another study, in which CAD was diagnosed on the basis of coronary CT angiography and patients were stratified according to Framingham Risk Score (FRS), demonstrated that miR-17-5p, miR-21-5p, miR-210-3p, miR-29b-3p, miR-7-5p, and miR-99a-5p were upregulated by greater than twofold in groups with CAD compared with those without, pointing out again the role of miRNA profiling in early detection of CAD [44]. 

Since MI is among the most common causes of HF, He and et al. investigated the association of two miRNAs, miR-328 and miR-134, with the acute phase of MI and their relationship with increased risk of mortality or development of HF during follow-up [45]. Indeed, the plasma levels of both miRNAs were found to be increased in 359 MI patients, when compared to 30 healthy subjects. Interestingly, further analyses indicated that miR-328 and miR-134 had a good predictive value as indicators of higher mortality and risk of developing post-MI HF. Thus, these two miRNAs were indicated as important biomarkers for the initial diagnosis and prognosis of negative complications following MI. Unfortunately, the authors failed to indicate the number of patients experiencing either HF onset or early mortality during MI follow-up; thus, no specific assessment could be done for the association of circulating miRNAs and the presence of asymptomatic LV dysfunction. 

In the case of high-risk patients with hypertension, hyperlipidemia, obesity, and diabetes mellitus, miRNAs could provide additional indications concerning the status of the cardiovascular system [46]. For instance, the plasma levels of miR-122 and miR-370 were found to be increased in patients with hyperlipidemia compared with controls and were associated with the presence and severity of CAD, proving their putative biomarker potential for risk stratification [46,47].

Another interesting field of research is the use of miRNAs as a marker of cardiotoxicity in oncologic patients undergoing chemotherapy or immunotherapy. Gioffrè et al. have already suggested to use plasma miRNAs as biomarkers of cardiac dysfunction onset upon specific anthracyclines treatment [48]. In their manuscript, they identified a cluster of circulating miRNAs (miR-122-5p, miR-499a-5p, and miR-885-5p) as predictors of doxorubicin-induced cardiac damage which could possibly develop into cardiotoxicity and/or HF. Despite being a pilot study, this investigation introduced, for the first time, the concept of miRNA-based selection of potentially cardiac-harmful drugs before their actual administration. In a way, the identified miRNAs could be indicators of a predisposition to HF onset upon doxorubicin treatment due to the synergy between cancer presence and genetic and/or epigenetic background. Similarly, a previous work identified miR-1 as a circulating biomarker in the setting of doxorubicin-induced reduction of LVEF in 56 breast cancer patients who showed late cardiotoxicity onset [49]. Furthermore, the plasmatic levels of miR-1254 and miR-579 were found to be significantly higher in patients diagnosed with bevacizumab-induced cardiotoxicity compared to those of healthy bevacizumab-treated controls [50]. 

Lastly, despite the recent improvement in radiation techniques, chest radiation can still be responsible for radiation-induced heart disease. Several miRNAs have been found to be up- or downregulated in patients who underwent radiotherapy. However, their association with radiation-induced heart disease remains to be elucidated. For instance, miRNA-29a levels were found to be reduced in patients treated with thoracic radiotherapy, and the reduction was proportional to the dose of radiotherapy. Therefore, this finding suggests the negative impact of radiation on vascular inflammation [51]. 

Further clinical investigations are needed to fully understand and exploit the potential of miRNAs as pathophysiology-based diagnostic markers for the identification of cardiac disease in its early asymptomatic phases.

### 3.2. Long Non-Coding RNAs 

Long non-coding RNAs (lncRNAs) are over 200-nucleotide long and are classified, according to their genomic location and polarity, in intronic, intergenic, bidirectional, sense, and antisense [52]. The potential use of lncRNAs as future biomarkers for HF is eliciting an increasing interest because they are differentially regulated during HF progression, are easily detectable and quantifiable in blood samples, and are more stable than proteins [53,54,55]. 

Among the currently identified lncRNAs, CDKN2B-AS1 (ANRIL) represses the transcription of the genes in the *INK4* locus, thus it is likely to play a role in inflammation [54]. Even if its transcription increases in HF, ANRIL has not been evaluated as a biomarker in a large population yet [54]. Similarly, the Myosin Heavy-Chain-Associated RNA Transcripts (MHRT) levels are higher in HF than in non-HF individuals [56,57]. To date, published data about MHRT sensitivity in discriminating asymptomatic individuals are uncertain [56,57]. Furthermore, Sirtuin 1 (Sirt1) long non-coding RNA binds to Sirt1 mRNA mediating its stabilization and the increase of protein level [58]. Currently, the effects of Sirt1 protein expression on HF development seem contrasting: some studies suggest that Sirt1 protein promotes hypertrophy, whereas others indicate reduction of both hypertrophy and fibrosis [53,58]. Patients with reduced ejection fraction (HFrEF) have a higher Sirt1 protein concentration than patients with preserved ejection fraction (HFpEF). However, in this model, differences in Sirt1 expression were not able to discriminate healthy controls from HFpEF [53]. Taken together, these data still question the potential of Sirt1 lncRNA in discriminating HFpEF patients. 

A number of studies have investigated the role of lncRNAs in cardiac physiology and pathology, identifying a set of new candidate biomarkers. β secretase-1 antisense (BACE1-AS) lncRNA stabilizes β secretase-1 (BACE1) mRNA leading to cardiomyocytes and endothelial cells cytotoxicity in HF [59]. To date, the eligibility of BACE1-AS as a biomarker in HF has not been investigated yet. Similarly, recent data indicate the involvement of the antisense lncRNAs CTBP1-AS2 and VIM-AS1 in HF comorbidities beside their known role in cancer pathogenesis [60]. Specifically, CTBP1-AS2 mediates cardiomyocyte hypertrophy, whereas VIM-AS1 is responsible for fibrosis [61,62]. Therefore, their pathological role in HF might be plausible but remains to be confirmed.

The aforementioned data suggest that antisense lncRNA use for early identification of asymptomatic LV dysfunction, although warranted, is still uncertain, and further studies on large populations are needed.

An interesting work from Kumarswamy et al. on transcriptomic analyses conducted in plasma RNA from MI patients showed that the circulating levels of the lcnRNA uc022bqs.1, named LIPCAR lncRNA, were found to predict adverse cardiac remodeling following MI. These results, beside showing the possibility to reproducibly detect lncRNAs in the plasma, indicated LIPCAR as a good candidate prognostic biomarker for HF upon ischemic events [63].

A very recent cross-sectional study was conducted on post-AMI patients either developing or not HF symptoms within 72 h from revascularization by percutaneous coronary intervention (PCI). In this setting, the blood levels of lncRNA necrosis-related factor NRF (lncRNA-NRF) were shown to be increased in AMI patients with HF compared with AMI patients without HF and had a good predictive value for the diagnosis of HF. The authors calculated the diagnostic potential for HF of lncRNA-NRF, obtaining an AUC value of 0.975, while the same analysis conducted for N-terminal pro-brain natriuretic peptide resulted in an AUC of 0.720. These findings seem to suggest that lncRNA-NRF may represent a marker of risk for the development of HF post-AMI, although the study was conducted on a limited population (n = 134) and HF onset was not evaluated longer than 72 h post PCI in non-HF patients [64].

A relatively new class of ncRNA is represented by circular RNAs (circRNA). These molecules consist of a circular, single-strand RNA lacking both 5′ and 3′ free termini and presenting a higher stability. CircRNAs are generated from back-splicing events in which a downstream 5′ splice donor joins an upstream 3′ splice acceptor and can be composed either of multiple exons or of a mix of exons and introns. CircRNAs have several and different molecular functions, similarly to all components of the ncRNA family, the most common consisting in acting as microRNA sponges, regulating transcription and splicing, and acting as a molecular adaptor for protein–protein interactions and ribosomal RNA processing [65]. Despite their importance, circRNA are not abundant but are present in all kinds of cells and tissues, including blood, where they can also be used as biomarkers. In this regard, one very interesting candidate is represented by circRNA MICRA, which was indicated by Vausort et al. as a predictor of LV dysfunction after MI, based on its expression in peripheral blood samples 3 to 4 months post reperfusion [66].

## 4. Exosomes

Exosomes are extracellular vesicles (EV) of 30–150 nm which bear a cargo consisting of DNA, RNA (including mRNA, miRNA, rRNA, lncRNA, PIWI-interacting RNAs, transfer RNAs, mitochondrial RNAs, Y RNAs, and vault RNAs), lipids, and proteins [67,68]. Exosomes are secreted by all cell types as a means to maintain homeostasis via cell-to-cell communication [69]. In people at high risk of cardiac diseases, exosomes cargo exhibits maladaptive variations [70]. During HF, cardiomyocytes, endothelial cells, fibroblasts, immune cells, and smooth muscle cells are all exosome producers and recipients causing HF progression, but their role in apparent healing is still unexplored. For instance, studies on therapeutic approaches suggest that mesenchymal stem/stromal cells (MSCs) release exosomes whose content is likely to contribute to myocardial healing [71,72,73,74,75].

Because of the cell ability to modify their cargo for rapidly adapting to novel pathophysiological conditions, exosomes might have a relevant diagnostic significance [76]. Changes in exosomes cargo have seemed to occur prior to the increase of HF traditional biomarkers, suggesting that exosomes monitoring could result in earlier detection of HF [71]. Specifically, among RNA subtypes in EV, exosomal miRNA (exo-miRNAs) evaluation seems promising for the search of new biomarkers. Increased levels of exo-miRNA-192, exo-miRNA-194, and exo-miRNA-34a were detected around 18 days after the acute event [77]. The role of all these exo-miRNAs in the p53 pathway mediating cardiomyocytes apoptosis warrants their involvement in HF pathogenesis [78,79]. Although these observations support the possible use of exo-miRNA-192, exo-miRNA-194, and exo-miRNA-34a for early HF diagnosis, their clinical evaluation is still lacking. In line with this, an increase in both extracellular vesicle release and cardiac-specific miRNAs (miR-1, miR-24, miR-133a/b, and miR-210) were documented to occur in the plasma of patients undergoing coronary artery bypass–graft surgery [80]. Intriguingly, although cardiac Troponin-I did not significantly correlate with cardiac miRNAs, it positively correlated with the plasma exosome level and exosomal cardiac miRNAs. To date, many other exo-miRNAs involved in HF initiation and progression have been identified [69,81]. Specifically, it has been shown that exo-miRNA-21-3p, exo-miRNA-132, exo-miRNA-200 participate in cardiac hypertrophy, whereas exo-miRNA-29 family members mediate fibrosis, and exo-miRNA-146a is causative of angiogenesis alterations [69]. However, none of them has been evaluated for diagnostic feasibility. On the contrary, even though its role in HF is still unknown and it is upregulated in patients with HF, exo-miRNA-92a has been proposed as a clinical biomarker because of its upregulation in patients with HF, high sensitivity/sensibility, and correlation with echocardiographic indices for LV dilation and dysfunction [82]. It is noteworthy that a recent study calls into question the exosomal origin of miRNA-92a, warranting further investigations [83]. Overall, the confirmation of miRNAs presence in exosomes together with the knowledge of their function in HF pathogenesis are emerging as important starting points for the clinical translation of exo-miRNAs.

Besides miRNAs, exosomes are also enriched in proteins. Some of them are common to almost all exosomes regardless of cell type or conditions because biogenesis-related, whereas other proteins are pathophysiological state-specific [69]. Proteins on the exosome surface allow the correct interaction of the EV with target cells, while protein cargo affects the activity of recipient cells [70,84]. Published data indicate that protein cargo influences the development of cardiovascular diseases [85,86]. In HF, heat shock protein 60 and 90 (Hsp60, Hsp90) participate in cardiomyocyte death and cardiac fibrosis, respectively [69]. Furthermore, exosomes contain tumor necrosis factor-alpha (TNF-α), which causes cardiomyocyte death, as well as angiotensin-converting enzyme (ACE) and Angiotensin II (Ang-II), which elicit hypertrophy [69,70]. The previously cited exosomal proteins have been suggested as biomarkers; nevertheless, their diagnostic value has not been appraised yet. A valuable and very promising exception is the work of Castellani et al., who analyzed plasma-derived EV surface protein profiles as a biomarker for the early diagnosis of cardiac allograft rejection. In their work, authors built and validated in an external cohort of patients a diagnostic model based on EV markers that were differentially expressed in controls and patients undergoing either antibody-mediated or acute cellular rejection [87]. The model was able to identify patients who underwent rejection with an accuracy of 86.5%.

Exosomes are present in different biological fluids and are stable in various conditions of temperature and pH [76,88,89]. Therefore, they seem suitable for maintaining their characteristics unchanged until sample preparation and analysis. 

Besides the promising results that suggest exosome suitability for diagnostic purposes, several limitations hamper their use in clinical practice [73]. In particular, the majority of the current data are based on a high variety of pre-clinical treatments and isolation/analytical procedures which are not officially recognized for clinical use [76]. Despite current data being still inconclusive in defining the value of exosomes in clinical practice, the theoretical potential of exosomes supports further clinical evaluation to determine their role as biomarkers.

## 5. Promising New Biomarkers 

**Figure 2:**
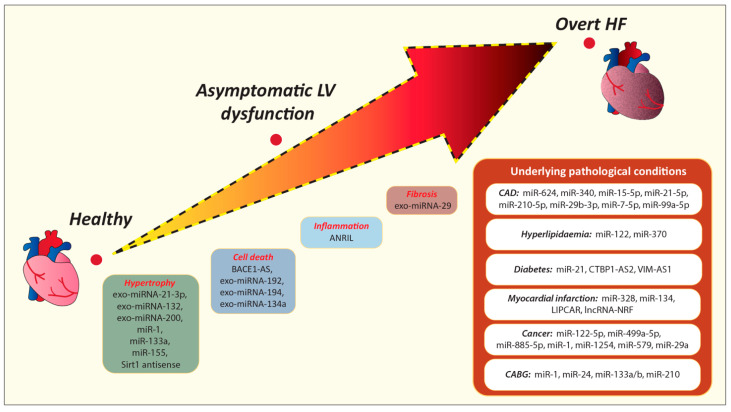
Promising new biomarkers that could be useful in the early detection of underlying pathophysiological processes that could lead to cardiac deterioration over time. CABG, Coronary artery bypass graft; CAD, Coronary artery disease; HF, Heart failure; LV, Left ventricular.

The previously cited miRNA, lncRNA, and exosomes seem promising biomarkers for understanding patients’ conditions and predicting cardiac deterioration. This opportunity is even more tempting for “apparently healed” and NYHA I patients. Pathologic conditions such as CAD, hypertension, and diabetes cause myocardial damage and increase the likelihood of HF development. The aforementioned data suggest miRNAs involved in CAD (e.g., miR-624, miR-340, miR-15-5p, miR-21-5p, miR-210-5p, miR-29b-3p, miR-7-5p, miR-99a-5p), diabetes (e.g., miR-21), and hyperlipidemia (e.g., miR-122, miR-370) as promising biomarkers (Figure 2) (Table 2). Similarly, antisense lncRNA such as CTBP1-AS2 and VIM-AS1 are associated with a higher incidence of diabetes, proving their potential as prognostic and diagnostic biomarkers. However, further clinical studies are needed to evaluate the prognostic power of these non-coding RNAs in the timely recognition of cardiac deterioration at its initial stage among asymptomatic individuals.

HF is a progressive disorder involving cellular, interstitial, and molecular changes, causing inflammation and pathological remodeling [90]. Alterations in metabolic pathways and hypoxia cause cardiomyocyte death and trigger subsequent alterations. BACE1-AS and exosomes cargo, consisting of exo-miRNA-192, exo-miRNA-194, and exo-miRNA-134a and, might be indicators of cardiomyocyte death. In order to remove damaged cells, the inflammatory response is activated, and ANRIL could be used as one of the indicators of inflammation, given the fact that this specific process influences ANRIL expression. For adapting to myocardial injury, the renin–angiotensin system stimulates protein production in fibroblasts and cardiomyocytes, contributing to fibrosis and hypertrophy [90]. Fibrosis consists in the accumulation of collagen, entailing increased myocardial stiffness and systolic and diastolic dysfunction [90]. The interstitial changes might be identified by early using Ang II, as well as exo-miRNA-29, contained in exosomes. Similarly, we deem that exosome cargo consisting of exo-miRNA-21-3p, exo-miRNA-132, and exo-miRNA-200 and non-coding RNAs such as miR-1, miR-133a might be promising indicators for the early detection of hypertrophy.

Noteworthy, the cohort of “apparently healed” patients describes a group of patients that have been diagnosed with cardiac disease but are asymptomatic and show signs of “reverse remodeling” due to the optimization of pharmacological therapy, that, as indicated by guidelines for HF treatment, relies on ACE inhibitors, beta-blockers, and aldosterone antagonists for their ability to reduce myocardial remodeling [90]. Interesting results were obtained among patients with DCM in whom, after symptom resolution and cardiac function recovery due to the therapy, the discontinuation of treatment led to worsening of the clinical condition [9]. In the same context, it is worth mentioning the category of NYHA I individuals, who could suffer the worsening of conditions over time because of the ongoing remodeling processes that continue to occur in their heart. Previously suggested miRNAs, antisense lncRNAs, and exosomes could be promising biomarkers in patients’ monitoring also in these circumstances, revealing the beginning and/or worsening of existing fibrosis and hypertrophy [39]. However, further investigation is needed to determine their exact role in pathophysiological pathways. 

Given the complexity of HF, it is unlikely that relying on a single prognostic factor would suffice to satisfy unmet needs in the diagnosis of individuals with HF. Hence, a multimarker approach including biomarkers involved in various pathophysiological processes might represent a promising opportunity for an early identification of patients at risk of HF. Achieving these goals would be a radical step forward in personalized medicine.

## 6. Conclusions

The identification of asymptomatic individuals at risk of developing HF would be an amazing achievement in terms of reduction in HF morbidity and mortality, as well as for the economy, given the fact that patients with HF impose a significantly high economic burden. However, most evidence concerning the use of biomarkers is related to overt cardiac diseases, and further studies are needed regarding their possible informative character in asymptomatic individuals. In the same context, non-coding RNA and exosomes cargo are paving their way in the cardiology field as biomarkers, providing insight into undergoing processes that could lead to HF. Therefore, further investigations should evaluate the expression levels of those new markers in the general population, define cut-offs, and identify their dynamic changes in pathophysiological conditions, which later on could be useful in detecting asymptomatic phases at the very beginning of the morbidity.

## Figures and Tables

**Table 1:** Traditional and emerging protein biomarkers, considering their advantages and disadvantages. BNP, B-type or brain natriuretic peptide; cTn, cardiac troponin; Gal-3, Galactin-3; NPs, Natriuretic peptides; NT-proBNP, N-terminal pro-B-type natriuretic peptide; sST2, soluble suppression of tumorigenicity 2.

Traditional Biomarkers			
Biomarkers	Advantages	Disadvantages	Ref.
**BNP, NT-proBNP**	− Higher values in asymptomatic individuals than healthy subjects	− Influenced by age, body mass index, and other conditions (e.g., renal failure, inflammatory pulmonary disease)	[10,14]
**Emerging Protein Biomarkers**			
Biomarkers	Advantages	Disadvantages	Ref.
**cTn**	− Highly sensitive detection	− Difficult definition for cut-offs − Influenced by age, gender, body max index, systolic pressure, diabetes mellitus	[19,20,22]
**sST2**	− Higher values in asymptomatic individuals than healthy subjects − Low biological variation vs NPs − Suitable for follow-up	− Useful only in combination with other NPs	[24,25]
**Gal-3**	− Higher in pre-symptomatic individuals	− Low specificity − Useful only in combination with other NPs	[26,27]
**Ghrelin**	− Differentiation according to HF stage	− Useful only in combination with other NPs	[28,29]

**Table 2:** Summary of miRNAs suggested as possible biomarkers. AMI, acute myocardial infarction; CAD, coronary artery disease; miRNA, micro-RNA; LV, Left ventricular.

Condition		miRNA	Ref
**LV diastolic dysfunction**	Hypertrophy	miR-1, miR-133a, miR-155, miR-21 exo-miRNA-21-3p, exo-miRNA-132, exo-miRNA-200	[39] [40] [69]
	Fibrosis	exo-miRNA-29	[69]
	Cell death	exo-miRNA-192, exo-miRNA-194, exo-miRNA-134a	[77]
**Chronic heart failure**		miR-221, miR-21, miR-409-5p, miR-376a, miR-154	[41]
**AMI**		miR-499 miR-328, miR-134	[42] [45]
**CAD**		miR-624, miR-340 miR-17-5p, miR-21-5p, miR-210-3p, miR-29b-3p, miR-7-5p, miR-99a-5p	[43] [44]
**Hyperlipidaemia**		miR-122, miR-370	[46,47]
**Medical treatment**		miR-122-5p, miR-499a-5p, miR-885-5p miR-1 miR-1254, miR-579	[48] [49] [50]
**Radiotherapy**		miRNA-29a	[51]

## Abbreviations

ACE	angiotensin-converting enzyme
AMI	acute myocardial infarction
Ang-II	angiotensin II
ATP	atrial natriuretic peptide
BACE1	β secretase-1
BACE1-AS	β secretase-1 antisense
BNP	B-type or brain natriuretic peptide
CAD	coronary artery disease
CHF	chronic heart failure
cTn	cardiac troponin
DCM	dilated cardiomyopathy
EV	extracellular vesicles
exo-miRNAs	exosomal miRNAs
FRS	Framingham Risk Score
Gal-3	Galectin-3
HF	heart failure
HFpEF	heart failure with preserved ejection fraction
HFrEF	heart failure with reduced ejection fraction
Hsp60	heat shock protein 60
Hsp90	heat shock protein 90
lncRNAs	long non-coding RNAs
LV	left ventricular
MI	myocardial infarction
miRNA	micro-RNA
MSCs	mesenchymal stem/stromal cells
NPs	natriuretic peptides
NT-proBNP	N-terminal pro-B-type natriuretic peptide
NYHA	New York Heart Association
sST2	soluble suppression of tumorigenicity 2
TNF-α	tumor necrosis factor-alpha
